# The Generalizability of Randomized Controlled Trials of Self-Guided Internet-Based Cognitive Behavioral Therapy for Depressive Symptoms: Systematic Review and Meta-Regression Analysis

**DOI:** 10.2196/10113

**Published:** 2018-11-09

**Authors:** Lorenzo Lorenzo-Luaces, Emily Johns, John R Keefe

**Affiliations:** 1 Department of Psychological and Brain Sciences Indiana University Bloomington Bloomington, IN United States; 2 Department of Psychology University of Pennsylvania Philadelphia, PA United States

**Keywords:** depression, psychotherapy, CBT, internet-based therapy, pharmacotherapy, generalizability, exclusion criteria, cognitive therapy, telemedicine, drug therapy, patient selection

## Abstract

**Background:**

Self-guided internet-based cognitive behavioral therapies (iCBTs) for depressive symptoms may substantially increase accessibility to mental health treatment. Despite this, questions remain as to the generalizability of the research on self-guided iCBT.

**Objective:**

We sought to describe the clinical entry criteria used in studies of self-guided iCBT, explore the criteria’s effects on study outcomes, and compare the frequency of use of these criteria with their use in studies of face-to-face psychotherapy and antidepressant medications. We hypothesized that self-guided iCBT studies would use more stringent criteria that would bias the sample toward those with a less complex clinical profile, thus inflating treatment outcomes.

**Methods:**

We updated a recently published meta-analysis by conducting a systematic literature search in PubMed, MEDLINE, PsycINFO, and EMBASE. We conducted a meta-regression analysis to test the effect of the different commonly used psychiatric entry criteria on the treatment-control differences. We also compared the frequency with which exclusion criteria were used in the self-guided iCBT studies versus studies of face-to-face psychotherapy and antidepressants from a recently published review.

**Results:**

Our search yielded 5 additional studies, which we added to the 16 studies identified by Karyotaki and colleagues in 2017. Few self-guided iCBT studies excluded patients with severe depressive symptoms (6/21, 29%), but self-guided iCBT studies were more likely than antidepressant (14/170, 8.2%) studies to use this criterion. However, self-guided iCBT studies did not use this criterion more frequently than face-to-face psychotherapy studies (6/16, 38%). Beyond this, we found no evidence that self-guided iCBTs used more stringent entry criteria. Strong evidence suggested that they were actually less likely to use most entry criteria, especially exclusions on the basis of substance use or personality pathology. None of the entry criteria used had an effect on outcomes.

**Conclusions:**

A conservative interpretation of our findings is that the patient population sampled in the literature on self-guided iCBT is relatively comparable with that of studies of antidepressants or face-to-face psychotherapy. Alternatively, studies of unguided cognitive behavioral therapy may sample from a more heterogeneous and representative patient population. Until evidence emerges to suggest otherwise, the patient population sampled in self-guided iCBT studies cannot be considered as less complex than the patient population from face-to-face psychotherapy or antidepressant studies.

## Introduction

### Background

Depression is one of the leading causes, if not the leading cause, of disability worldwide [1,2]. A well-recognized barrier to reducing the level of disability associated with depression and other common mental disorders is the scarcity of available treatment providers [3]. Although there is a scarcity of providers, the research data support a wide variety of treatments for depression that vary in their modality (eg, self-management, psychotherapy, medications), as well as in how time and resource intensive they are for patients and providers. The combination of antidepressants and psychotherapy is widely seen as providing the best cost-benefit ratio for severe depression [4]. However, it is nearly impossible to advocate this or any other treatment approach as a first-line intervention because depression is highly heterogeneous in its prognosis [5-7]. For example, 50% to 75% of patients with first-onset major depressive episodes recover within a 3- to 6-month period, but 15% to 20% of cases are chronic [8-11]. Similarly, although 50% of patients recover and do not experience a relapse in 20- to 30-year follow-ups, approximately 35% of individuals experience a recurrent course [9,12,13]. Moreover, the presentation of depressive symptoms is highly heterogeneous. Approximately half of cases are classified as mild to moderate [14], but a proportion of cases involve psychotic symptoms [15] or suicidal risk (eg, 27%-53% [16]). As well, there is a substantial degree of comorbidity with other disorders [14].

A major advancement in the study of treatments for depression is the discovery that self-guided internet-based cognitive behavioral therapy (iCBT) can be more effective than control conditions [17]. In these interventions, patients complete interactive Web-based programs based on the principles of cognitive behavioral therapy with no therapeutic support, although sometimes technical support is available. The efficacy of these interventions can be enhanced by therapist support, although this effect may be smaller than previously thought (eg, standardized mean difference symptoms = 0.27 [18-20]). The high prevalence of depression and the ubiquity of internet access and mobile phone ownership make self-guided iCBTs hugely promising in reducing the burden of disability associated with depressive symptoms, even if they were somewhat less effective than guided iCBTs [21]. Kazdin and Blase [3] have called attention to the fact that the number of individuals with mental disorders such as depression far outnumbers the number of mental health providers available to deliver treatment. Thus, self-guided iCBT has the potential for a greater public health impact than guided iCBT or other forms of treatment that require contact with a trained professional [21]. However, a key concern in evaluating the self-guided iCBT treatment literature is the degree to which the patient population in randomized controlled trials (RCTs) is representative of the heterogeneous nature of the prognosis, severity, and comorbidity found in depressed patients. More than 20 years ago, Seligman [22] pointed out that the literature attesting to the efficacy of face-to-face psychotherapy was limited by virtue of the inclusion and exclusion criteria used, which excluded patients with subthreshold symptoms as well as comorbid conditions. Westen and Morrison [23] quantified the nature of this problem, reporting that most (68%) patients with depression were excluded from a typical psychotherapy study because of suicidality, comorbid disorders, especially substance use, and subclinical symptoms. Furthermore, these authors reported that the number of participants excluded from psychotherapy trials was related to the study-level effect size, such that studies that excluded more patients tended to find larger effect sizes for psychotherapy (see also van der Lem et al [24]). Zimmerman et al [25] made similar observations in the literature on antidepressants. More recently, Zimmerman and colleagues [26-29] reported on the inclusion and exclusion (henceforth *entry*) criteria for RCTs of antidepressant medications. They stated that most studies excluded patients on the basis of a minimum symptom threshold, suicidality, psychotic features, and substance use disorders.

### Objective

We sought to review the entry criteria used in trials of self-guided iCBT. We did this by updating and coding the studies from the latest meta-analysis of self-guided iCBT, which was published by Karyotaki et al [17]. In addition to reporting the overall frequency with which specific criteria were used, we sought to explore the relationship between the specific entry criteria and the study-level outcome. Finally, to provide some context for evaluating the frequency with which self-guided iCBT studies used different exclusion criteria, we compared the frequency of use of entry criteria in self-guided iCBT trials versus the rate of inclusion criteria used in face-to-face psychotherapy trials and trials of antidepressant medications. The aim of this analysis was for face-to-face psychotherapy and antidepressant studies to serve as a kind of benchmark against which to compare the self-guided iCBT literature. A prior study suggested that face-to-face psychotherapy trials were less likely than antidepressant trials to use most of the specific entry criteria coded by the study authors [30]. Other studies similarly suggested that psychotherapy studies may be somewhat more generalizable than pharmacotherapy studies for adolescent depression [31], borderline personality disorder [32], social anxiety [33], and posttraumatic stress disorder [34], although not generalized anxiety disorder [35]. Based on prior data [23], we hypothesized that the use of more stringent entry criteria would be associated with better outcomes. However, because studies of self-guided iCBT are often fully remote and lack human support, we hypothesized that studies of self-guided iCBT would use more stringent psychiatric entry criteria than would face-to-face psychotherapy and pharmacotherapy trials.

## Methods

### Identification and Rating of Studies

We obtained RCTs exploring the efficacy of self-guided iCBT for depression in adults by referencing and updating the latest meta-analysis of self-guided iCBT for depression [17]. Those authors consulted a broad database of psychological treatments for depression that was constructed from a systematic literature search of free terms combining “psychotherapy” and “depression” in PubMed, EMBASE, PsycINFO, and the Cochrane Library published up to January 1, 2016. We updated the review by applying the same search criteria (see Multimedia Appendix 1), narrowing our search for internet-based interventions. We included studies if they included adults (>18 years of age) with a diagnosis of major depressive disorder on a psychiatric semistructured interview, with elevated symptoms of depression (ie, any specific cutoff score on a depression questionnaire), or who were seeking or undergoing treatment for depression. Two of the authors (LLL, EJ) rated all articles for the presence of common psychiatric inclusion and exclusion criteria used in treatment studies [26], resolving discrepancies by consensus. Descriptive analyses summarizing the specific entry criteria of the iCBT trials are presented. We rated risk of bias according to the Cochrane Collaboration risk-of-bias assessment tool [36]. We rated the primary outcome studies. When we could not determine a specific domain category from the main outcome study, we rated protocol articles if they were available or any publicly available study registry (eg, ClinicalTrials.gov).

### Meta-Regression

We calculated Hedges *g* to quantify the difference between self-guided iCBT and control conditions on symptoms of depression. Hedges *g* is derived from the difference between the average posttreatment scores on self-reported measures of depressive symptoms in the 2 groups (ie, self-guided iCBT group vs the control group), divided by the pooled standard deviation while adjusting for small-sample bias. For entry criteria that had sufficient representation in the dataset (ie, ≥3 studies used them), we conducted meta-regressions, using the R statistical computing language version R-3.5.0. (R Foundation) package metafor [37], to examine the relationship between using specific entry criteria and outcomes. First, we conducted individual meta-regressions in which we regressed the study-level effect size on each of the exclusion criteria used. Then, we regressed the number of exclusion criteria used on the study-level outcomes. Finally, we conducted a simultaneous meta-regression in which we regressed outcomes on all entry criteria.

Meta-regressions were run in a random effects meta-analytic framework, using exclusion criteria either as dummy-coded categorical variables (0/1) or as an ordinal variable (ie, the number of exclusions). In line with current best practices for calculating confidence intervals with more accurate coverage and less-inflated type I error, we used the Sidik-Jonkman random effects estimator and the Hartung-Knapp adjustment [38,39].

### Benchmarking Against Antidepressant and Face-to-Face Psychotherapy

To provide a rough index of how entry criteria for guided iCBT studies compare with those of other treatments, we drew on recent reviews of the entry criteria of trials of adult depression with antidepressants [26-29] and face-to-face psychotherapy [30]. These reviews employed searches in PubMed, EMBASE, and PsycINFO, as well as individual meta-analyses and specific journals, to identify acute treatment outcomes studies for depression. The search was limited to studies in which antidepressants or face-to-face psychotherapy was compared with a control condition, so studies of multiple treatments (eg, 2 psychotherapies) were only included if a control condition was used. There are systematic differences between antidepressant and face-to-face pharmacotherapy studies regarding the type of controls employed [40]. Virtually all pharmacotherapy studies use a pill placebo, whereas the efficacy of face-to-face psychotherapy, as well as self-guided iCBT, is tested with a more diverse mix of controls, including a waiting list, treatment as usual, pill placebos, and other conditions (eg, relaxation) that are intended as a control for nonspecific effects (eg, attention). Thus, we searched for face-to-face psychotherapy trials in which a waiting list, treatment as usual, or placebo control was used. We excluded trials if they focused exclusively on comorbidities (eg, only depression and alcohol use), whether psychiatric or general medical, as they are, by definition, less inclusive. As well, we did not include trials focused on subtypes of depression, inpatients, or patients with specific symptom profiles (eg, cognitive symptoms). The application of these criteria yielded 170 studies of antidepressants and 16 studies of face-to-face psychotherapy. To compare the differences between the self-guided iCBT studies and studies of antidepressants and face-to-face psychotherapies, we used a chi-square test, or Fisher exact test when we expected any cell to have a frequency lower than 5.

## Results

### Study Characteristics

**Figure 1:**
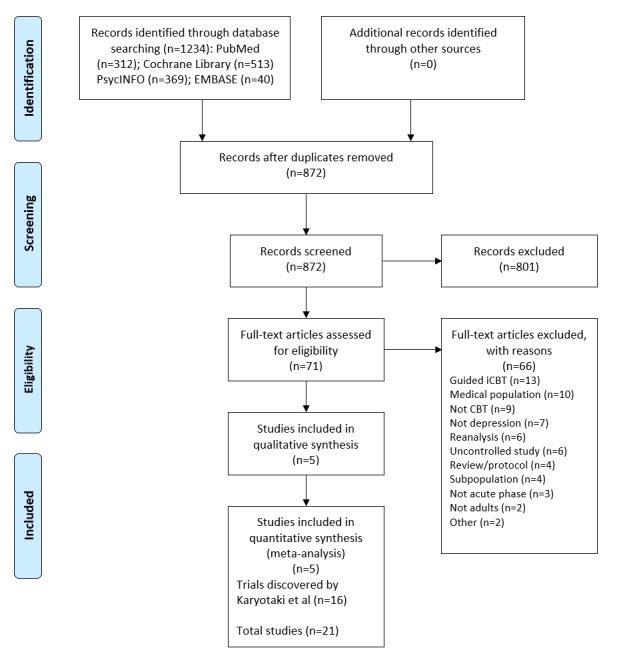
Preferred Reporting Items for Systematic Reviews and Meta-Analyses (PRISMA) flowchart for self-guided internet-based cognitive behavioral therapy (iCBT) studies included in the systematic review and meta-regression analysis. CBT: cognitive behavioral therapy.

We identified 5 new studies (see Multimedia Appendix 1) since the publication of the review by Karyotaki et al [17] that met our inclusion criteria (see Figure 1). Thus, we coded 21 RCTs comparing self-guided iCBTs versus a control in our updated review. These studies analyzed data for 4781 participants. Like Karyotaki et al, we found risk of bias to be low across most studies (see Multimedia Appendix 1).

There were differences between the self-guided iCBT studies, the antidepressant studies, and the face-to-face psychotherapy studies in terms of sample size per study arm (*F*_2,204_=6.45, *P*=.002) and country of origin (χ^2^_1_=48.3, *P*<.001), but no differences in the percentage of studies that excluded participants based on an upper age limit (χ^2^_1_=1.5, *P*=.48). Antidepressant (mean 120.69, SD 74.10) and self-guided iCBT (mean 140.61, SD 109.16) studies tended to have more participants in each study arm than face-to-face psychotherapy studies did (mean 55.12, SD 39.48). Most self-guided iCBT studies originated from single sites within European countries (14/21, 67%). By way of contrast, only 5 of the psychotherapy studies were conducted in Europe (5/16, 31%) and, instead, the face-to-face psychotherapy studies were more likely to be published in the United States (8/16, 50%). Very few antidepressants studies were published from within single European countries (15/170, 8.8%), as most were published in the United States (101/171, 59.4%).

Table 1 lists the psychiatric inclusion and exclusion criteria used in the self-guided iCBT trials.

**Table 1:** Psychiatric exclusion criteria of randomized controlled trials of self-guided internet-based cognitive behavioral therapy for depressive symptoms (N=21).

Exclusion criteria	n (%)
Severity scale score below the cutoff (minimum score)	15 (71)
Severity scale score above the cutoff (maximum score)	6 (29)
Psychosis	12 (57)
Substance abuse or dependence	5 (24)
Significant suicidal ideation or risk	13 (62)
History of suicide attempt(s)	1 (5)
Episode length too long	1 (5)
Episode length too short	1 (5)
Any axis II disorder	0 (0)
Any axis I disorder	1 (5)
Borderline personality disorder	0 (0)
Antisocial personality disorder	0 (0)
Schizotypal personality disorder	0 (0)

At least half of the iCBT studies used the following 3 criteria: psychotic disorder or current psychotic symptoms (12/21, 57%), a minimum symptom severity on a depression scale (15/21, 71%), and significant suicidal ideation (13/21, 62%). The other criteria were used relatively infrequently. As has been noted of studies of antidepressants and face-to-face psychotherapy, there was considerable variability in the operationalization of these criteria. For example, of the 15 studies that excluded patients on the basis of a minimum score on a depression severity measure, 6 used the Patient Health Questionnaire-8 or -9 as an exclusion criterion, 4 used the Beck Depression Inventory (BDI), and 2 used the Kessler Psychological Distress Scale. The remaining 3 studies used other scales (eg, the Center for Epidemiologic Studies Depression Scale [CES-D]).

### Effects of Entry Criteria on Outcomes

Only 5 of the exclusionary criteria were used with enough frequency (ie, >3 uses) that we could explore whether their use was associated with treatment outcomes: minimum symptom severity, maximum symptom severity, psychosis, substance misuse, and suicidality. Table 2 lists the results of the meta-regressions in which we regressed the study-level effect sizes in the 5 exclusionary criteria, individually as well as considered simultaneously. The updated meta-analysis found results similar to those by Karyotaki et al [17]. At the study level, self-guided iCBT was associated with more improvement in depression than were the control conditions (*g*=0.33, 95% CI 0.20-0.46, SE 0.06; *P*<.001).

We detected a high degree of between-study heterogeneity of effect sizes (I^2^=76%), indicating that exclusion criteria could potentially explain between-studies differences in effects. Despite this, the meta-regressions did not find any significant effects of specific exclusionary criteria (*P* values >.39) or the total number of criteria used on study-level treatment outcomes (B=–0.01, 95% CI –0.09 to 0.10, SE 0.05; *P*=.92). The study-level exclusion criterion that appeared to have the largest effect on study-level differences was the use of a minimum of symptom severity as an exclusion (B=–0.12, 95% CI –0.41 to 0.17, SE 0.14; *P*=.39). A simultaneous regression considering all of the exclusion criteria in tandem likewise did not find any significant effect of the use of specific entry criteria on outcomes (*P* values >.28).

### Comparison of Entry Criteria in Internet-Based Cognitive Behavioral Therapies, Antidepressants, and Psychotherapy

Table 3 shows the comparisons of the frequency with which different entry criteria were used in studies of self-guided iCBT versus studies of face-to-face psychotherapy versus studies of antidepressant medications. Contrary to our hypothesis, virtually all the exclusion criteria coded were used less frequently in the iCBT trials than in face-to-face psychotherapy and antidepressant trials, though not all these differences were statistically significant.

When specifically compared with studies of antidepressant medications, studies of self-guided iCBT were less likely to use almost all the exclusion criteria coded. For example, compared with studies of antidepressants (143/170, 84.1%), self-guided iCBT studies were less likely (12/21, 57%) to exclude patients on the basis of psychotic symptoms or a diagnosis (χ^2^_1_=7.2, *P*=.01). Similarly, 41.2% (70/170) of studies on antidepressants excluded patients on the basis of borderline personality disorder, although no self-guided iCBT study used this criterion (Fisher *P*<.001). The only exception to this general pattern of self-guided iCBT studies being more, rather than less, inclusive was on the basis of a maximum symptom severity exclusion. Only 29% (6/21) of the self-guided iCBT studies excluded patients on the basis of high symptom severity. However, this exclusion occurred more frequently in the iCBT studies than in the antidepressant studies (14/170, 8.2%; χ^2^_1_=6.2, *P*=.01). The self-guided iCBT studies were no more likely than face-to-face psychotherapy studies (6/16, 38%; χ^2^_1_=.05, *P*=.83) to use this criterion.

**Table 2:** Meta-regression coefficients for the relationship between individual exclusion criteria and study-level internet-based cognitive behavioral therapy controlled outcomes. No findings were significant at *P*<.05.

Exclusion criteria	Single-predictor models, *g* (95% CI)	Simultaneous-predictor model, *g* (95% CI)
Minimum symptom severity	–0.12 (–0.41 to 0.17)	–0.18 (–0.52 to 0.16)
Maximum symptom severity	–0.04 (–0.33 to 0.25)	–0.10 (–0.45 to 0.26)
Psychosis	0.07 (–0.20 to 0.34)	0.04 (–0.38 to 0.46)
Substance problems	0.02 (–0.29 to 0.33)	0.05 (–0.39 to 0.49)
Suicidality	0.09 (–0.18 to 0.36)	0.16 (–0.24 to 0.55)

**Table 3:** Comparison of the frequency of use of different inclusion and exclusion criteria across self-guided internet-based cognitive behavioral therapy (iCBT), face-to-face psychotherapy (F2F), and antidepressant medication (AM) trials.

Exclusion criteria	iCBT (n=21)	F2F (n=16)	AM (n=170)	*P* value		
				iCBT vs F2F vs AM	iCBT vs F2F	iCBT vs AM
Minimum symptom severity	15 (71)	13 (81)	170 (100.0)	<.001	.70	<.001
Maximum symptom severity	6 (29)	6 (38)	14 (8.2)	<.001	.83	.01
Psychosis	12 (57)	14 (88)	143 (84.1)	.02	.07	.01
Substance abuse or dependence	5 (24)	12 (75)	137 (80.6)	<.001	<.001	<.001
Suicidal risk	13 (62)	9 (56)	128 (75.3)	.14	.99	.29
Prior suicide attempt(s)	1 (5)	2 (12)	35 (20.6)	.21	.57	.13
Episode length too long	1 (5)	2 (12)	34 (20.0)	.21	.57	.13
Episode length too short	1 (5)	2 (12)	81 (47.6)	<.001	.57	<.001
Any axis II disorder	0 (0)	3 (19)	60 (35.3)	<.001	.07	<.001
Any axis I disorder	1 (5)	1 (6)	46 (27.1)	.01	>.99	.03
Borderline personality disorder	0 (0)	5 (31)	70 (41.2)	<.001	.01	<.001
Antisocial personality disorder	0 (0)	5 (31)	68 (40.0)	<.001	.01	<.001
Schizotypal personality disorder	0 (0)	5 (31)	63 (37.1)	<.001	.01	<.001

Most of the differences between self-guided iCBT and face-to-face psychotherapy studies did not meet the statistical significance threshold of *P*<.05, although the arithmetic differences were often in favor of the self-guided iCBT studies. Face-to-face therapy studies were more likely to specifically exclude participants based on the diagnoses of borderline, schizotypal, or antisocial personality disorder (each 5/16, 31%) than were the iCBT studies, which did not use this exclusion (each 0/21, 0%; Fisher *P*<.001).

By far the largest observed difference between the iCBT studies and the face-to-face and antidepressants studies was that iCBT studies infrequently excluded patients on the basis of a substance use disorder (5/21, 24%), though this exclusion was typical in antidepressant (137/170, 80.6%; Fisher *P*<.001) and face-to-face psychotherapy studies (12/16, 75%; Fisher *P*=.003).

## Discussion

### Principal Findings

We summarized the exclusion criteria used in self-guided iCBT studies, explored the relationship of the use of these criteria and the treatment effect size reported in the trial, and compared the frequencies with which such criteria were used in antidepressant trials or trials of face-to-face psychotherapy. Overall, self-guided iCBT studies infrequently used exclusion criteria that are the norm in studies of depression. Contrary to our hypotheses, we did not find the type and number of exclusion criteria to be related to outcomes. Also contrary to our hypothesis, self-guided iCBT trials were either equally likely or less likely to use specific psychiatric entry criteria.

### Limitations

A noteworthy limitation of this review is the small number of self-guided iCBT studies. It is possible that the entry criteria used had a small effect on outcomes, but a larger number of studies is needed to detect this effect. Our review was limited to studies of self-guided iCBTs. While these interventions may have a broader public health impact than guided iCBT [21], there is evidence that they have somewhat smaller effects and lower rates of treatment completion [18-20]. Future research should study the entry criteria in studies of guided iCBT.

The fact that studies did not use a specific entry criterion (eg, substance use disorder) does not imply that the trial actually had patients representative of that feature, which is another limitation of our study. Because studies do not uniformly report on all characteristics of their patient sample (eg, substance use, duration, suicide risk), it is impossible to test for the presence of these features of the sample across all the studies. It is also possible that studies used specific entry criteria but did not disclose them in the published report. Moreover, the psychiatric inclusion and exclusion criteria are only one aspect of the generalizability of the sample. Self-guided iCBT studies may have more selective patient samples by virtue of other criteria that they use explicitly (eg, access to the internet) or implicitly (eg, willingness to participate in an iCBT study).

While we updated the review by Karyotaki et al [17], we drew on published reviews by Lorenzo-Luaces et al [30], instead of updating this review as well. A difference between the 2 reviews is that the review by Lorenzo-Luaces and colleagues was limited to studies in which patients had a diagnosis of major depressive disorder. This difference specifically limited the number of face-to-face therapy studies eligible for coding. Our aim, however, was not to provide an exact estimate of the difference in frequency of use of exclusion criteria, but to provide a benchmarking context to evaluate the frequency with which self-guided iCBT studies excluded patients. The studies also differed by year of publications, as per the search strategy, country of origin, and size. These differences, along with the differences we identified as per our objectives, suggest that the studies are not directly or indirectly comparable (eg, as in a network meta-analysis). Our aim, however, was not to claim that the evidence base behind self-guided iCBTs is equivalent to the evidence base behind antidepressants or face-to-face psychotherapy but to provide a frame of reference against which to compare the entry criteria of self-guided iCBTs.

### Implications

Only 29% of the self-guided iCBT studies excluded patients on the basis of severe depression, but it is still worth noting that self-guided iCBT studies appeared more likely than antidepressant studies to exclude participants on this basis. The perception that self-guided iCBT will not be effective for cases of more severely symptomatic depression aligns with common sense but is not supported by research data. For example, Bower et al [41] reported that the effects of self-guided internet-based therapies were more rather than less pronounced among patients high in symptom severity. In our analyses, we found no evidence that the effects of iCBT varied strongly according to exclusions by high or low symptom severity. It is possible that self-guided iCBTs are less effective for patients who have more complex presentations, with symptom severity being only one index of case complexity [5]. For example, the presence of anxiety and chronic depression duration have all been implicated in treatment outcomes in depression and may relate to lower response to iCBT [42]. Although we did not find the use of psychosis as an exclusion criterion to be related to outcomes, psychotic depression is relatively rare, and even studies allowing these patients into the trial may have had a low representation of psychotic depression. In contrast, approximately 50% of cases of depression are rated as severe or very severe. It is probable that patients self-select into treatment trials in a way that patients with severe depression avoid iCBT studies, but the data do not strongly support this conclusion. For example, Karyotaki et al [17] reported a mean score of 28 on the BDI for patients in their dataset along with a score of 26 on the CES-D, both of which are in the moderate to severe range for each measure.

Self-guided iCBT studies often excluded patients on the basis of suicidal risk. Our findings suggest that this exclusion criterion was not associated with outcomes, a finding that mirrors the results of van der Lem et al [24], as well as the fact that suicidal risk is not consistently reported as a predictor of treatment outcomes with face-to-face psychotherapy or antidepressants. A common exclusion criterion in antidepressant and face-to-face psychotherapy studies is substance use disorder, although this was missing from most self-guided iCBT studies. The findings of van der Lem et al [24] suggested that alcohol use disorder is not a strong predictor of outcomes in treatment for depression, and other studies have also not found an association between substance use disorder and outcomes in treatment for depression [43]. Given that there is no evidence to suggest negative outcomes for suicidal patients and those with substance use disorders, as well as the low rate of treatment utilization in these and other patient groups, more self-guided iCBT studies specifically recruiting from these patient groups are needed. Hoertel et al [44] have argued that using, or not using, specific criteria may influence not only the external validity and generalizability of a study but also its internal validity, by virtue of influencing outcomes. This runs counter to the prevailing logic that there is a trade-off between external and internal validity. Overall, our results suggest that the inclusion criteria commonly used in the field sacrifice external validity but provide no gains in internal validity.

### Conclusions

To our knowledge, this is the first exploration of the effects of inclusion and exclusion criteria in self-guided iCBT studies, as well as the first comparison of the specific inclusion and exclusion criteria used in RCTs of iCBTs versus studies of face-to-face psychotherapy or antidepressants. Our findings can be taken to suggest that self-guided iCBT studies are more inclusive by design than studies of antidepressants or face-to-face psychotherapy. It is possible that, by using remote designs in which no individual face-to-face interviews can be conducted, self-guided iCBT trials limit their ability to exclude participants on specific features. For example, no self-guided iCBT trial excluded participants due to personality pathology broadly construed or borderline, schizotypal, or antisocial personality diagnosis, which are usually measured in psychiatric interviews, not self-reported questionnaires. By comparison, around a third of psychotherapy and around 40% of antidepressant trials used these exclusions. Given the differences in the entry criteria used, it stands to reason that, by design, studies of self-guided iCBT may be characterized by a more heterogeneous group of patients than studies of antidepressant or face-to-face psychotherapy. This level of heterogeneity in the underlying patient population increases the external validity of the research. Moreover, this variability can facilitate the discovery of process-outcome correlations [45], as well as effects of individual patient differences on outcomes [5]. This heterogeneity in the patient population may also contribute to heterogeneity in the overall effect size reported in treatment-control comparisons. Until evidence emerges to the contrary, however, it cannot be said that iCBT studies apply more stringent inclusion and exclusion criteria than studies of other treatments for depression or that the efficacy of the treatments is inflated by using a less complex or severe patient population.

## Appendix

Multimedia Appendix 1 List of studies, risk-of-bias assessments, and example search terms.

## Abbreviations

BDI: Beck Depression Inventory
CES-D: Center for Epidemiologic Studies Depression Scale
iCBT: internet-based cognitive behavioral therapy
RCT: randomized controlled trial
