# Supplementary material for: The Generalizability of Randomized Controlled Trials of Self-Guided Internet-Based Cognitive Behavioral Therapy for Depressive Symptoms: Systematic Review and Meta-Regression Analysis
Source: J Med Internet Res. 2018 Nov 9;20(11):e10113. doi: 10.2196/10113 (PMC6251981; doi:10.2196/10113)
Supplement: Multimedia Appendix 1 [file jmir_v20i11e10113_app1.pdf]

Appendix:

1 - List of included studies

2 - Risk of bias assessment

3 – Example search terms

Studies identified by Karyotaki et al. 2017:

1. Berger T, Hammerli K, Gubser N, Andersson G, Caspar F. Internet-based treatment of depression: a randomized controlled trial comparing guided with unguided self-help. Cognitive behaviour therapy. 2011;40(4):251-266.
2. Christensen H, Griffiths KM, Jorm AF. Delivering interventions for depression by using the internet: randomised controlled trial. BMJ (Clinical research ed). 2004;328(7434):265.
3. de Graaf LE, Gerhards S, Arntz A, et al. Clinical effectiveness of online computerised cognitive–behavioural therapy without support for depression in primary care: randomised trial. The British Journal of Psychiatry. 2009;195(1):73-80.
4. Farrer L, Christensen H, Griffiths KM, Mackinnon A. Internet-based CBT for depression with and without telephone tracking in a national helpline: randomised controlled trial. PloS one. 2011;6(11):e28099.
5. Gilbody S, Littlewood E, Hewitt C, et al. Computerised cognitive behaviour therapy (cCBT) as treatment for depression in primary care (REEACT trial): large scale pragmatic randomised controlled trial. BMJ (Clinical research ed). 2015;351:h5627.
6. Kleiboer A, Donker T, Seekles W, van Straten A, Riper H, Cuijpers P. A randomized controlled trial on the role of support in internet-based problem solving therapy for depression and anxiety. Behaviour research and therapy. 2015;72(6):63-71.
7. Klein JP, Berger T, Schröder J, et al. Effects of a Psychological Internet Intervention in the Treatment of Mild to Moderate Depressive Symptoms: Results of the EVIDENT Study, a Randomized Controlled Trial. Psychotherapy and psychosomatics. 2016;85(4):218-228.
8. Meyer B, Berger T, Caspar F, Beevers CG, Andersson G, Weiss M. Effectiveness of a novel integrative online treatment for depression (Deprexis): randomized controlled trial. Journal of medical Internet research. 2009;11(2):e15.
9. Meyer B, Bierbrodt J, Schröder J, et al. Effects of an Internet intervention (Deprexis) on severe depression symptoms: Randomized controlled trial. Internet Interventions. 2015;2(1):48-59.
10. Moritz S, Schilling L, Hauschildt M, Schroder J, Treszl A. A randomized controlled trial of internet-based therapy in depression. Behaviour research and therapy. 2012;50(7-8):513-521.
11. Phillips R, Schneider J, Molosankwe I, et al. Randomized controlled trial of computerized cognitive behavioural therapy for depressive symptoms: effectiveness and costs of a workplace intervention. Psychol Med. 2014;44(4):741-752.
12. Spek V, Nyklíček I, Smits N, et al. Internet-based cognitive behavioural therapy for subthreshold depression in people over 50 years old: a randomized controlled clinical trial. Psychological medicine. 2007;37(12):1797-1806.
13. Clarke G, Reid E, Eubanks D, et al. Overcoming depression on the Internet (ODIN): a randomized controlled trial of an Internet depression skills intervention program. Journal of medical Internet research. 2002;4(3):e14.
14. Clarke G, Eubanks D, Reid CK, et al. Overcoming Depression on the Internet (ODIN)(2): a randomized trial of a self-help depression skills program with reminders. Journal of medical Internet research. . 2005;7(2):e16.
15. Clarke G, Kelleher C, Hornbrook M, DeBar L, Dickerson J, Gullion C. Randomized effectiveness trial of an Internet, pure self-help, cognitive behavioral intervention for depressive symptoms in young adults. Cognitive behaviour therapy. 2009;38(4):222-234.

16. Mira A, Bretón-López J, Garcia-Palacios A, Quero S, Baños RM, Botella C. An internet-based program for depressive symptoms using human and automated support: a randomized control trial. Neuropsychiatr Dis Treat. In press.

New studies identified by systematic review:

17. Terides, M. D., Dear, B. F., Fogliati, V. J., Gandy, M., Karin, E., Jones, M. P., & Titov, N. (2018). Increased skills usage statistically mediates symptom reduction in self-guided internet-delivered cognitive–behavioural therapy for depression and anxiety: a randomised controlled trial. Cognitive behaviour therapy, 47(1), 43-61.
18. Montero-Marín, J., Araya, R., Pérez-Yus, M. C., Mayoral, F., Gili, M., Botella, C., ... & Nogueira-Arjona, R. (2016). An internet-based intervention for depression in primary Care in Spain: a randomized controlled trial. Journal of medical Internet research, 18(8).
19. Arean, P. A., Hallgren, K. A., Jordan, J. T., Gazzaley, A., Atkins, D. C., Heagerty, P. J., & Anguera, J. A. (2016). The use and effectiveness of mobile apps for depression: results from a fully remote clinical trial. Journal of medical Internet research, 18(12).
20. Beevers, C. G., Pearson, R., Hoffman, J. S., Foulser, A. A., Shumake, J., & Meyer, B. (2017). Effectiveness of an internet intervention (Deprexis) for depression in a United States adult sample: A parallel-group pragmatic randomized controlled trial. Journal of consulting and clinical psychology, 85(4), 367.
21. Berger, T., Krieger, T., Sude, K., Meyer, B., & Maercker, A. (2017). Evaluating an e-mental health program ("deprexis") as adjunctive treatment tool in psychotherapy for depression: Results of a pragmatic randomized controlled trial. Journal of affective disorders, 227, 455-462.

Cochrane Risk of Bias Assessment:

Study	Judgement	Support
**Arean et al 2016**		

Study	Judgement	Support
Random sequence generation (selection bias)	Low risk	We randomly assigned participants to 1 of the 3 apps using a random number generator built into the eligibility survey.
Allocation concealment (selection bias)	Low risk	Main article and protocol study do not directly reference allocation concealment but participants in this study were randomly assigned to treatment and this was a fully remote trial in which there was "minimal contact with study staff."
Blinding of participants and personnel (performance bias)	N/A	It is not possible blind participants to treatment condition
Blinding of outcome assessment (detection bias)	N/A	(Self-reported outcome)
Incomplete outcome data (attrition bias)	Low risk	Effect sizes computed from ITT analyses.
Selective reporting (reporting bias)	Low risk	No obvious deviations from published protocol.
Other bias	Low risk	A conflict of interest is reported in regards to one of the conditions the iCBT is being compared against. No obvious source of bias.
**Montero-Marin et al 2016**		
Random sequence generation (selection bias)	Low risk	"Participants were individually randomized using blocked random number sequence. Randomization to one of the three groups. Blocks were administered in each of the regions, using a computer-generated random number sequence."
Allocation concealment (selection bias)	Low risk	"A person who had no other involvement in the study managed the random allocation to groups. This procedure was implemented through a remote central telephone line. The sequence was concealed until all individuals had been randomized."
Blinding of participants and personnel (performance bias)	N/A	It is not possible blind participants to treatment condition
Blinding of outcome assessment (detection bias)	N/A	(Self-reported outcome)
Incomplete outcome data (attrition bias)	Low risk	Effect sizes computed from ITT analyses.
Selective reporting (reporting bias)	Low risk	No obvious deviations from published protocol.
Other bias	Low risk	No obvious source of bias.
**Berger et al. 2018**		
Random sequence generation (selection bias)	Low risk	"automated computer generated random numbers"
Allocation concealment (selection bias)	Low risk	"table placed in a secured web-based database and was concealed to the investigators and the therapists."
Blinding of participants and personnel	N/A	It is not possible blind participants to treatment condition

Study	Judgement	Support
(performance bias)		
Blinding of outcome assessment (detection bias)	N/A	(Self-reported outcome)
Incomplete outcome data (attrition bias)	Low risk	Effect sizes computed from ITT analyses.
Selective reporting (reporting bias)	Low risk	Due to recruitment problems and resource limitations, were unable to recruit number of participants originally proposed. Study sufficiently powered to detect a medium-sized effect of intervention.
Other bias	Low risk	Study co-author employed by company who owns iCBT intervention. No obvious source of bias.
**Beevers et al. 2017**		
Random sequence generation (selection bias)	Low risk	"Randomization was conducted with an allocation schedule of random numbers that was created by a computerized random number generator."
Allocation concealment (selection bias)	Low risk	"Participants who were deemed eligible were placed on this list by a research assistant (J.H.) who did not conduct telephone interviews."
Blinding of participants and personnel (performance bias)	N/A	It is not possible blind participants to treatment condition
Blinding of outcome assessment (detection bias)	N/A	(Self-reported outcome)
Incomplete outcome data (attrition bias)	Low risk	Effect sizes computed from ITT analyses.
Selective reporting (reporting bias)	Low risk	No obvious source of bias.
Other bias	Low risk	Study co-author employed by company who owns iCBT intervention. No obvious source of bias.
**Terides et al., 2017**		
Random sequence generation (selection bias)	Low risk	"A permuted block randomisation sequence was used, utilising blocks of eight (four treatment group, four control group), through the website www.randomizer.org."
Allocation concealment (selection bias)	Low risk	"...both the experimenter and the applicants were blinded to this allocation [sequence]"
Blinding of participants and personnel (performance bias)	N/A	It is not possible blind participants to treatment condition
Blinding of outcome assessment (detection bias)	N/A	(Self-reported outcome)
Incomplete outcome data (attrition bias)	Low risk	Effect sizes computed from ITT analyses.
Selective reporting (reporting bias)	Low risk	No obvious source of bias.

Study	Judgement	Support
Other bias	Low risk	No obvious source of bias.

Example search terms for Pubmed:

(Internet [All Fields] OR web [All Fields] OR “internet-based” [All Fields] or “web-based” [All Fields] AND Psychotherapy [MH] OR psychotherap*[All Fields] OR cbt[All Fields] OR "behavior therapies"[All Fields] OR "behavior therapy"[All Fields] OR "behavior therapeutic"[All Fields] OR "behavior therapeutical"[All Fields] OR "behavior therapeutics"[All Fields] OR "behavior therapeutist"[all Fields] OR "behavior therapeutists"[All Fields] OR "behavior treatment"[All Fields] OR "behavior treatments"[All Fields] OR "behaviors therapies"[All Fields] OR "behaviors therapy"[All Fields] OR "behaviors therapeutics"[All Fields] OR "behaviors therapeutic"[All Fields] OR "behaviors therapeutical"[All Fields] OR "behaviors therapeutist"[All Fields] OR "behaviors therapeutists"[All Fields] OR "behaviors treatment"[All Fields] OR "behaviors treatments"[All Fields] OR "behavioral therapies"[All Fields] OR "behavioral therapy"[All Fields] OR "behavioral therapeutics"[All Fields] OR "behavioral therapeutic"[All Fields] OR "behavioral therapeutical"[All Fields] OR "behavioral therapeutist"[All Fields] OR "behavioral therapeutists"[All Fields] OR "behavioral treatment"[All Fields] OR "behavioral treatments"[All Fields] OR "behaviour therapies"[All Fields] OR "behaviour therapy"[All Fields] OR "behaviour therapeutic"[All Fields] OR "behaviour therapeutical"[All Fields] OR "behaviour therapeutics"[All Fields] OR "behaviour therapeutist"[all Fields] OR "behaviour therapeutists"[All Fields] OR "behaviour treatment"[All Fields] OR "behaviour treatments"[All Fields] OR "behaviours therapies"[All Fields] OR "behaviours therapy"[All Fields] OR "behaviours therapeutics"[All Fields] OR "behaviours therapeutic"[All Fields] OR "behaviours therapeutical"[All Fields] OR "behaviours therapeutist"[All Fields] OR "behaviours therapeutists"[All Fields] OR "behaviours treatment"[All Fields] OR "behaviours treatments"[All Fields] OR "behavioural therapies"[All Fields] OR "behavioural therapy"[All Fields] OR "behavioural therapeutics"[All Fields] OR "behavioural therapeutic"[All Fields] OR "behavioural therapeutical"[All Fields] OR "behavioural therapeutist"[All Fields] OR "behavioural therapeutists"[All Fields] OR "behavioural treatment"[All Fields] OR "behavioural treatments"[All Fields] OR "cognition therapies"[All Fields] OR "cognition therapie"[All Fields] OR "cognition therapy"[All Fields] OR "cognition therapeutical"[All Fields] OR "cognition therapeutic"[All Fields] OR "cognition therapeutics"[All Fields] OR "cognition therapeutist"[All Fields] OR "cognition therapeutists"[All Fields] OR "cognition treatment"[All Fields] OR "cognition treatments"[All Fields] OR psychodynamic[All Fields] OR Psychoanalysis[MH] OR psychoanalysis[All Fields] OR psychoanalytic*[All Fields] OR counselling[All Fields] OR counseling[All Fields] OR Counseling[MH] OR "problem-solving"[All Fields] OR mindfulness[All Fields] OR (acceptance[All Fields] AND commitment[All Fields]) OR "assertiveness training"[All Fields] OR "behavior activation"[All Fields] OR "behaviors activation"[All Fields] OR "behavioral activation"[All Fields] OR "cognitive therapies"[All Fields] OR "cognitive therapy"[All Fields] OR "cognitive therapeutic"[All Fields] OR "cognitive therapeutics"[All Fields] OR "cognitive therapeutical"[All Fields] OR "cognitive therapeutist"[All Fields] OR "cognitive therapeutists"[All Fields] OR "cognitive treatment"[All Fields] OR "cognitive treatments"[All Fields] OR "cognitive restructuring"[All Fields] OR (("compassion-focused"[All Fields] OR "compassion-focussed"[All Fields]) AND (therapy[SH] OR therapies[All Fields] OR therapy[All Fields] OR therape*[All Fields] OR therapis*[All Fields]OR Therapeutics [OR treatment*[All Fields])) OR ((therapy[SH] OR therapies[All Fields] OR therapy [All Fields] OR therape*[All Fields] OR therapis*[All Fields] OR Therapeutics[MH] OR treatment*[All Fields]) AND constructivist*[All Fields]) OR "metacognitive therapies"[All Fields] OR "metacognitive therapy"[All Fields] OR "metacognitive therapeutic"[All Fields] OR "metacognitive therapeutics"[All Fields] OR "metacognitive therapeutical"[All Fields] OR "metacognitive therapeutist"[All Fields] OR "metacognitive therapeutists"[All Fields] OR "metacognitive treatment"[All Fields] OR

"metacognitive treatments"[All Fields] OR "meta-cognitive therapies"[All Fields] OR "meta-cognitive therapy"[All Fields] OR "meta-cognitive therapeutic"[All Fields] OR "meta-cognitive therapeutics"[All Fields] OR "meta-cognitive therapeutical"[All Fields] OR "meta-cognitive therapeutist"[All Fields] OR "meta-cognitive therapeutists"[All Fields] OR "meta-cognitive treatment"[All Fields] OR "meta-cognitive treatments"[All Fields] OR "solution-focused therapies"[All Fields] OR "solution-focused therapy"[All Fields] OR "solution-focused therapeutic"[All Fields] OR "solution-focused therapeutics"[All Fields] OR "solution-focused therapeutical"[All Fields] OR "solution focused therapies"[All Fields] OR "solution focused therapy"[All Fields] OR "solution focused therapeutic"[All Fields] OR "solution focused therapeutics"[All Fields] OR "solution focused therapeutical"[All Fields]OR "solution-focussed therapies"[All Fields] OR "solution-focussed therapy"[All Fields] OR "solution-focussed therapeutic"[All Fields] OR "solution-focussed therapeutics"[All Fields] OR "solution-focussed therapeutical"[All Fields]OR "solution focussed therapies"[All Fields] OR "solution focussed therapy"[All Fields] OR "solution focussed therapeutic"[All Fields] OR "solution focussed therapeutics"[All Fields] OR "solution focussed therapeutical"[All Fields] OR "self-control therapies"[All Fields] OR "self-control therapy"[All Fields] OR "self-control therapeutics"[All Fields] OR "self-control therapeutical"[All Fields] OR "self-control therapeutic"[All Fields] OR "self-control training"[All Fields] OR "self-control trainings"[All Fields] OR "self control therapies"[All Fields] OR "self control therapy"[All Fields] OR "self control therapeutics"[All Fields] OR "self control therapeutical"[All Fields] OR "self control therapeutic"[All Fields] OR "self control training"[All Fields] OR "self control trainings"[All Fields] AND (Depressive Disorder[MH] OR Depression[MH]OR dysthymi*[All Fields] OR "affective disorder"[All Fields]OR "affective disorders"[All Fields] OR "mood disorder"[All Fields] OR "mood disorders"[All Fields] OR depression*[All Fields] OR depressive*[All Fields] OR "dysthymic disorder"[MeSH Terms]) AND ((randomized controlled trial [pt] OR controlled clinical trial [pt] OR randomized [tiab] OR randomly [tiab] NOT (animals[mh] NOT (animals[mh] AND humans [mh]))
